# Prevalence of Diabetic Nephropathy among Patients with Type 2 Diabetes Mellitus in China: A Meta-Analysis of Observational Studies

**DOI:** 10.1155/2020/2315607

**Published:** 2020-02-03

**Authors:** Xin-Xin Zhang, Jun Kong, Ke Yun

**Affiliations:** ^1^Department of Ophthalmology Laboratory, The Fourth Affiliated Hospital of China Medical University, Shenyang 110006, China; ^2^Department of Laboratory Medicine, The First Affiliated Hospital of China Medical University, Shenyang 110001, China

## Abstract

**Background:**

Diabetic nephropathy (DN) is an important cause of end-stage renal disease and is recognized as a public health problem worldwide. However, there have been no nationwide surveys of DN prevalence in China. This study is aimed at estimating the pooled prevalence of DN among patients with type 2 diabetes in China.

**Methods:**

Published studies on the prevalence of DN among patients with type 2 diabetes published from January 1980 to October 2019 were systematically reviewed using PubMed, Embase, Google Scholar, Chinese Wanfang databases, and Chinese National Knowledge Infrastructure. The pooled prevalence of DN was estimated with the random effects model using R software. Prevalence estimates were also stratified by study design, methodological approach, and study population characteristics.

**Results:**

Thirty studies with a total of 79,364 participants were included in our study. The overall pooled prevalence of DN was 21.8% [95% confidence interval (CI): 18.5-25.4%]. Subgroup analysis found that the prevalence of DN varied significantly according to different DM and DN diagnostic criteria (*P* < 0.05); the pooling estimate was the highest in the west region of 41.3%, followed by that in the east region of China with 22.3%, northeast region with 20.7%, and central region with 15.6% (*P* < 0.05); the pooling estimate was the highest in the west region of 41.3%, followed by that in the east region of China with 22.3%, northeast region with 20.7%, and central region with 15.6% (*P* < 0.05); the pooling estimate was the highest in the west region of 41.3%, followed by that in the east region of China with 22.3%, northeast region with 20.7%, and central region with 15.6% (

**Conclusions:**

The prevalence of DN is high in Chinese patients with type 2 diabetes and shows geographic and gender variation. These data indicate that national strategies aimed at primary and secondary prevention of DN and screening programs for DN are urgently needed to reduce the risk and burden of DN in China.

## 1. Introduction

Diabetes mellitus (DM) is a worldwide public health challenge. WHO estimated that there were around 422 million people living with diabetes and that there was a rising trend in the number of people living with DM [1]. Among these people, type 2 diabetes (T2DM) accounts for over 90% of all persons with diabetes [2]. Diabetic nephropathy (DN) is frequently associated with T2DM and the leading cause of chronic kidney disease and end-stage renal disease [3]. Importantly, with the increasing incidence of T2DM, the frequency of DN has also increased [4]. Examining the prevalence and influencing factors of DN in patients with T2DM is, therefore, an important first step in understanding the disease burden and developing additional research priorities as well.

In China, with the rapid economic growth and urbanization, lifestyle changed significantly. At the same time, the prevalence of T2DM has been increasing dramatically. IDF Diabetes Atlas estimated that in 2017, the prevalence of diabetes was 10.9%, and it estimated that there were 114 million people living with diabetes and 61 million people with undiagnosed diabetes [5]. Besides, the national survey in China also showed that a large proportion of diabetes was undiagnosed and that patients with newly diagnosed diabetes accounted for 60% of the total diabetic population [6]. Consequently, it is striking that DN among those with T2DM has become one of the most important public health crises in China, and there is an urgent need to assess the epidemiological characteristics and risk factors of DN in T2DM in China to implement effective interventions.

Although the DN epidemic in China is striking [5], the prevalence and risk factors of DN among Chinese patients with T2DM have not been systematically studied nationwide, and the variation of DN prevalence in T2DM in China also has not yet been reported, which limits the ability to realize its severity and characteristics. Therefore, we conducted a meta-analysis of studies on DN to determine the national prevalence of DN and its variation in patients with T2DM in China.

## 2. Materials and Methods

### 2.1. Literature Search

This meta-analysis was conducted according to the PRISMA guideline. The PubMed, Embase, Google Scholar, Chinese Wanfang databases, and Chinese National Knowledge Infrastructure (CNKI) were searched. We used the following search terms: (“nephropathy” OR “kidney diseases”) AND (“diabetes mellitus” OR “diabetes” OR “mellitus”) AND (“epidemiology” OR “prevalence”). We searched for studies published from January 1980 to October 2019 to identify relevant articles. The literature was limited to those published in Chinese and English as both reviewers are fluent in these languages.

### 2.2. Study Selection and Data Extraction

Diabetes is a disease that blood glucose levels rise higher than normal and for extended periods. T2DM is the most common form of diabetes [7]. DN is a syndrome characterized by the presence of pathological levels of urinary albumin excretion, diabetic glomerular lesions, and loss of glomerular filtration rate (GFR) in diabetics [8]. In this meta-analysis, the definition and diagnostic criteria of this study were all taken from the included articles. We used the following inclusion and exclusion criteria. Studies were included in our meta-analysis if (1) included Chinese participants and (2) reported quantitative data regarding DN prevalence. Studies were excluded if (1) duplicated reports; (2) included patients with type 1 diabetes or other special populations, such as pregnant women; and (3) were studies that were qualitative or postintervention or included special professional people, such as doctors. When additional data were needed, we attempted to contact the authors to obtain relevant data.

Two investigators (KY and XXZ) independently reviewed the search results and selected articles to determine eligibility and to extract study data. Disagreements of data extraction among two reviewers were reconciled by discussion. Standardized Excel spreadsheet abstraction forms were designed to capture all relevant information required for analyses, including first author, date of publication, diagnosis standard for DN, diagnosis standard for DM, study location, population source, urban/rural, age of subjects, BMI, sex, duration of DM (years), systolic pressure, diastolic pressure, number of patients with DM and DN, and quality score.

### 2.3. Quality Assessment

Methodological quality assessments were conducted using the Strengthening the Reporting of Observational Studies in Epidemiology (STROBE) checklist of observational studies [9]. Two authors (KY and XXZ) evaluated each article's quality based on the checklist, and discrepancies were addressed by discussion. Each of the items was categorized as yes (1 score) or no (0 score) to denote whether the study fulfillment of corresponding criteria. If an item was not applicable for that study design, it was scored as “not applicable” (NA). The methodological quality score of studies was grouped according to the mean of the total scores into lower than 20 points or equal or higher than 20 points for quality analysis.

### 2.4. Statistical Analyses

The pooled prevalence of DN was calculated using the inverse variance method, as previously described. Briefly, if the tests met the hypothesis of homogeneity, fixed effects models were used; otherwise, random effects models were used [10]. Heterogeneity across the included studies was analyzed using the *Q* test and the *I*^2^ index (values of 25%, 50%, and 75% are taken as low, medium, and high heterogeneity, respectively). Subgroup analyses were performed by the study year, diagnostic criteria for DN and DM, geographical areas, population source, sample size, age, BMI, sex, DM duration, study quality score, and blood pressure to explore the influence of potential heterogeneity factors on the pooling estimation. The geographic areas are divided according to the standard of the geographical division of China [11].

The leave-one-out sensitivity test was used to confirm that our findings were not driven by any single study. In addition, Egger's tests were used to detect potential publication bias by examining the funnel plot symmetry. *P* < 0.05 indicated statistical significance. Statistical analyses were performed using R software (Version 3.0.1.).

## 3. Results

### 3.1. Identification and Selection of Eligible Studies

**Figure 1:**
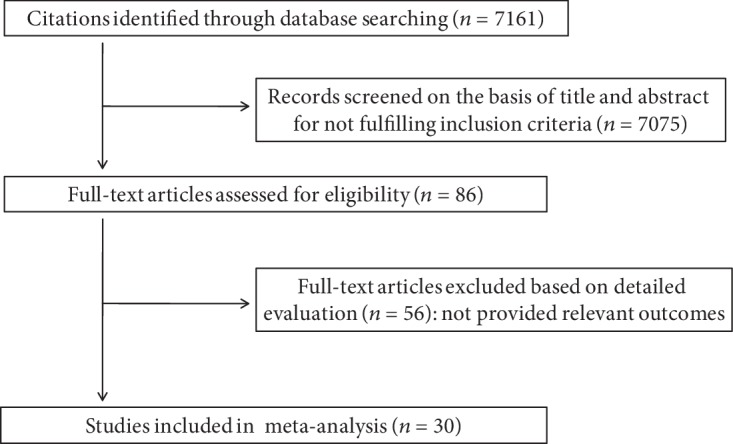
Flow diagram of studies included in meta-analysis.

A total of 7161 citations were retrieved in the literature search. Of these, 7075 were excluded after screening titles and abstracts, and 86 were selected for further evaluation. Finally, 30 articles that provided the rates of DN in adults with T2DM were included in this review (Figure 1).

A descriptive summary of the included studies is provided in Table 1. The included studies were conducted between 1991 and 2017 across 13 provinces/cities in China. All the included studies were cross-sectional studies. The sample size ranged from 46 to 31,574. Of the included studies, three were from the central region of China, nineteen from the east region, three from the northeast region, two from the west region, and two from Hong Kong. The study populations were from two different sources: five studies were community-based, whereas twenty-five were hospital-based. The mean participant age was 59.3 years, and the mean course of DM was 7.7 years.

### 3.2. Estimated Pooled Prevalence of DN in Chinese Adults with Type 2 Diabetes

**Figure 2:**
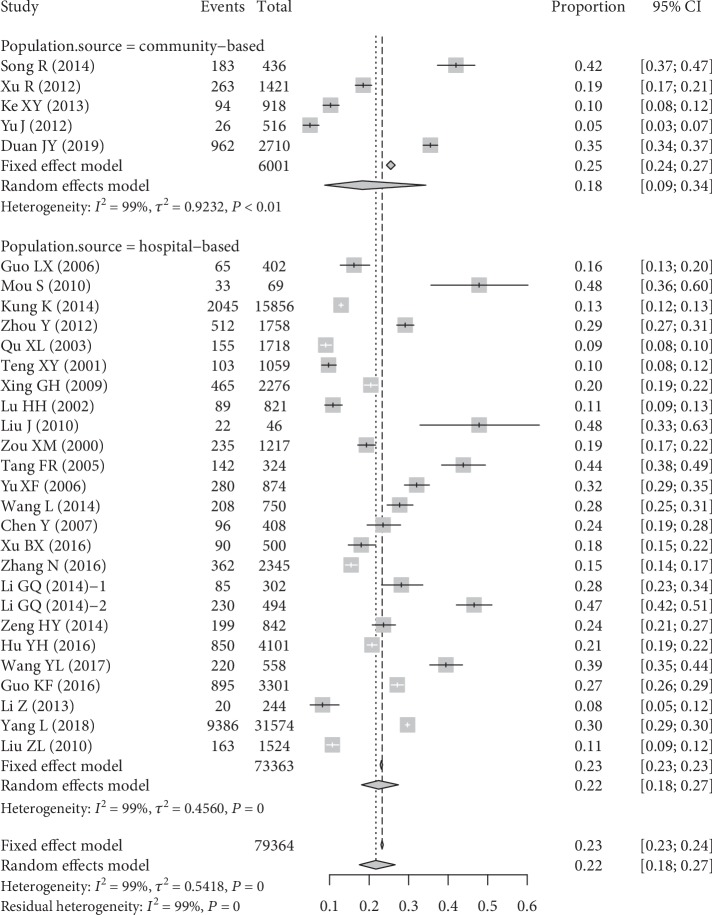
Forest plot displaying the pooled prevalence of DN in patients with type 2 diabetes in both population sources.

A total of 30 studies, including 79,364 adults with T2DM, were evaluated. Substantial heterogeneity across the included studies was observed (*I*^2^ = 99.1%, *Q* = 3103.46, *P* < 0.01). Therefore, random effects models were used, and the pooled prevalence of DN was 21.8% (95% CI: 18.5-25.4%) (Figure 2).

### 3.3. Subgroup Analysis

Table 2 shows the subgroup analyses of the prevalence of DN among participants with T2DM. Significant differences were found in the diagnostic criteria of DM and DN (*P* < 0.01), region (*P* < 0.01), and gender (*P* < 0.01). The prevalence of DN varied significantly according to different DM and DN diagnostic criteria; studies using the KDOQI 2014 diagnostic criteria for DN (39.4%) and confirmed DM by history of DM (35.3%) had the highest DN prevalence compared to that established using other standards. The pooled prevalence of DN in the west region of China of 41.3% (95% CI: 37.1-45.6%) was the highest, followed by that in the east region with 22.3% (95% CI: 18.6-26.5%), northeast region with 20.7% (95% CI: 15.2-27.6%), and central region with 15.6% (95% CI: 4.9-39.8%) (*P* < 0.01). The pooled prevalence rates of DN were higher in the male-dominated studies, 27.7% (95% CI: 24.1-31.7%), compared with the female-dominated studies, 17.6% (95% CI: 12.6-24.0%) (*P* < 0.01).

### 3.4. Difference between Locations

**Figure 3:**
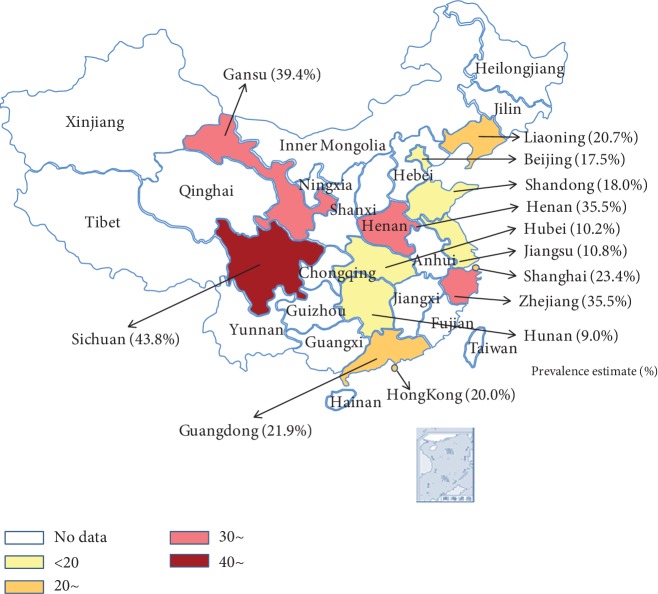
Regional distribution of pooled prevalence of DN among patients with type 2 diabetes.

To further understand regional differences, we performed stratified analyses by province/municipality. These analyses showed that the highest prevalence of DN was in the four provinces of Sichuan (43.8%), Gansu (39.4%), and Zhejiang and Henan (35.5%) provinces. Jiangsu (10.8%), Hubei (10.2%), and Hunan (9.0%) provinces had a low DN prevalence among patients with T2DM in China. The patterns of DN prevalence across the country are shown in Figure 3.

### 3.5. Quality Assessment, Sensitivity Analysis, and Publication Bias

**Figure 4:**
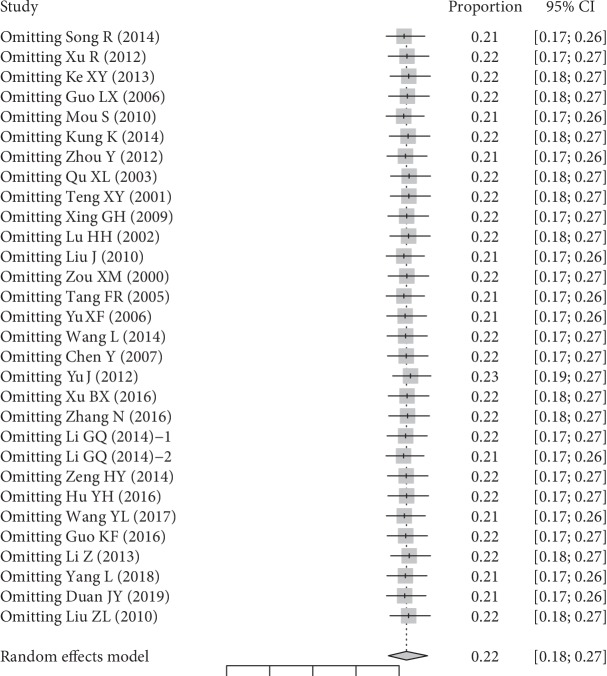
*k* − 1 leave-one-out sensitivity tests.

The mean (range) quality assessment score was 20 (12–26). Twenty-five studies had equal or higher than the mean strobe quality score (20 points) of all included studies, while 5 had lower than 20 points ([Supplementary-material supplementary-material-1]). In sensitivity analyses, the leave-one-out sensitivity tests revealed that no individual study influenced the total outcome (Figure 4). Egger's regression test of funnel plot asymmetry indicated that there was no potential publication bias among the included studies (*t* = −1.2966, *P* = 0.205) ([Supplementary-material supplementary-material-1]).

## 4. Discussion

To the best of our knowledge, the present study is the first meta-analysis to estimate the pooled prevalence of DN in people with T2DM in China, which included 30 studies with 79,364 patients with T2DM. The pooled prevalence of DN showed that nearly one-fifth of patients with diabetes might have nephropathy complications. The detailed estimates in this study showed that diabetes complicated with nephropathy is a serious public health challenge for the health care system and may result in a large social and economic burden in China. Our findings could help in relevant policy-making and planning and allocation of health care resources.

The pooled DN prevalence in our study was in agreement with a German study (20–30%) [41], but slightly lower than what was found in a cross-sectional population-based study among urban T2DM patients in south India (26.1%) [42]. However, the DN prevalence in our study was higher than that reported by a Saudi national diabetes registry-based study (10.8%) [43]. These phenomena may be explained by racial or ethnic differences in the prevalence of DN [44]. In China, the pandemic of DM, predominantly T2DM, is alarming [5]. Considering the delayed diagnosis of diabetes in China [45], DN would be an important social and economic burden. It should be paid more attention to develop mandatory measures for early detection and prevention. Several studies have proven that diet and exercise interventions seem to be effective methods for risk reduction for metabolic disorders. Early health screening, health education, and combination lifestyle therapies should be implemented in the high-risk population to reduce the disease burden for both individuals and society [46].

Subgroup analyses were performed to evaluate the impact of different stratifications on the prevalence of pooled DN. We found that DN prevalence varied significantly according to different diagnostic criteria for DM and DN. In fact, over the past 40 years, the diagnosis criteria of DM and DN have been changed several times, and different diagnostic criteria might influence the diagnosis and surveillance of DN [47]. This finding inspired us that the harm and benefit of different biomarkers and definitions for DM and DN on identification of cases, population prevalence estimation, and health costs should be evaluated. At the same time, the finding also suggests multicenter studies in the future with consistent methods and protocols for the identification of DM and DN.

We found that the pooled prevalence of DN in the west region was the highest and that further stratified analyses by province/municipality showed that Sichuan and Gansu were the provinces with the highest prevalence of DN. Similarly, a study in the United States showed that there was geographic variation in adjusted incident rates of end-stage renal disease [48]. The geographic difference in diabetes prevalence and detection in China is an important reason for geographic variation of DN prevalence among those with T2DM. Maigeng et al. [49] reported that the southwest had the lowest regional detection of diabetes of 15.6% (11.7, 20.5%) and thereby a delayed diagnosis of DN. Different healthy lifestyles, diet, and development of health care systems may also account for this difference [50], such as physical inactivity, control of hypertension, serum cholesterol control, and quitting smoking. Besides, environmental and genetic factors that might explain this phenomenon need further investigation [51] and indicating that more intervention resources of DM and DN should be put in the west of China. Meanwhile, the promotion of awareness of keeping a healthy lifestyle, diabetes prevention, and early medical intervention is still needed for the prevention of DN.

We also found that the pooled prevalence of DN was higher in the male-dominated studies than in the female-dominated studies, which echoed by the study of de Hauteclocque et al. [52]. However, some other studies found that females with T2DM had a higher risk of DN than males [53]. The inconsistent results regarding sex differences might have been caused by different risk factors with diabetes incidence and late diabetes diagnosis [54], and this finding could be furtherly explored in the future.

Our study had several limitations. First, most studies included in our study were hospital-based, which might have led to an overestimation of DN prevalence among the T2DM population because of referral bias. Thus, dichotomized outcomes according to population source (hospital-based and community-based) were both provided, and this should be considered in interpreting our results. Second, potential heterogeneous factors, such as the different diagnostic criteria for DM and DN, and variation of study sample size, might add heterogeneity of pooled prevalence estimation. To evaluate the influence, subgroup analyses and leave-one-out sensitivity analysis were both used to quantify the potential impact.

In conclusion, our results indicate that the prevalence of DN in China is high and shows geographic and gender variation. National strategies aimed at primary and secondary prevention, as well as a geographically targeted screening program for DN among participants with T2DM, are urgently needed to reduce the increasing burden of DN in China.

## Figures and Tables

**Table 1:** Summarized information of studies included in meta-analysis.

First author (publication year)	Survey date	Diagnosis standards for DN	Diagnosis standards for DM	Area	Population source	Age (years)	BMI	Sex (%males)	Course of DM (years)	Systolic blood pressure	Diastolic blood pressure	Sample size	Quality score
Song (2014) [12]	2012	Clinical diagnosis	Clinical diagnosis	Shanghai	Community-based	<59	25	55.7	NA	NA	NA	436	12
Xu (2012) [13]	2008-2009	KDOQI 2007	ADA criteria 2005	Shanghai	Community-based	61.3 ± 9.7	NA	40.8	7.9 ± 6.3	NA	NA	1421	22
Ke (2013) [14]	2011	Mogensen criteria	WHO criteria 1999	Huangshi, Hubei	Community-based	NA	NA	53.1	NA	NA	NA	918	20
Guo (2006) [15]	2002	Clinical diagnosis	WHO criteria 1999	Beijing	Hospital-based	51.1 ± 11.8	NA	60	NA	NA	NA	402	22
Mou (2010) [16]	2003-2008	Renal biopsy	History of DM	Shanghai	Hospital-based	53.1 ± 7.5	NA	52.2	NA	140.9 ± 26.3	85.1 ± 11.5	69	22
Kung (2014) [17]	2009-2011	Clinical diagnosis	Clinical diagnosis	Hong Kong	Hospital-based	60(20-)	25	48.8	7.3 ± 6.2	138 ± 18.3	76 ± 10.5	15856	22
Zhou (2012) [18]	2003-2010	Clinical diagnosis	WHO criteria 1999	Beijing	Hospital-based	60.8 ± 12.8	25	56.8	9.2 ± 7.5	NA	NA	1758	21
Qu (2003) [19]	1994-2001	Mogensen criteria	1985 WHO/1999 China criteria	Changsha, Hunan	Hospital-based	57.3 ± 21.3	NA	47.4	7.9 ± 4.3	NA	NA	1718	17
Teng (2001) [20]	1997-2000	Clinical diagnosis	WHO criteria 1985	Shanghai	Hospital-based	>43	NA	NA	NA	NA	NA	1059	22
Xing (2009) [21]	2007-2009	Clinical diagnosis	WHO criteria 1999	Benxi, Liaoning	Hospital-based	61.2 ± 11.0	NA	51.2	9.6 ± 2.8	NA	NA	2276	22
Lu (2002) [22]	1996-2001	Clinical diagnosis	Clinical diagnosis	Suzhou, Jiangsu	Hospital-based	>45	23 ± 3	NA	NA	NA	NA	821	21
Liu (2010) [23]	2003-2006	Renal biopsy	Clinical diagnosis	Shanghai	Hospital-based	53 ± 7.7	NA	56.5	6	NA	NA	46	21
Zou (2000) [24]	1993-1998	Clinical diagnosis	WHO criteria 1985	Beijing	Hospital-based	57.7 ± 15.0	NA	62.8	6.4 ± 7.4	NA	NA	1217	20
Tang (2005) [25]	NA	Clinical diagnosis	History of DM	Panzhihua, Sichuan	Hospital-based	17-83	NA	52.5	0-20	NA	NA	324	18
Yu (2006) [26]	1991-2000	Clinical diagnosis	History of DM	Hangzhou, Zhejiang	Hospital-based	59 ± 12	NA	49.3	6 ± 6	NA	NA	874	19
Wang (2014) [27]	2013	Clinical diagnosis	History of DM	Fushun, Liaoning	Hospital-based	59.2 ± 12	24.6 ± 3	45.1	7.1 ± 6.1	NA	NA	750	20
Chen (2007) [28]	2005	ADA criteria 1997	Clinical diagnosis	Shanghai	Hospital-based	60.3 ± 9.7	24.3 ± 3.3	48.1	5.4 ± 5.3	133.2 ± 17.6	77.9 ± 9.1	408	20
Yu (2012) [29]	2011	Clinical diagnosis	WHO criteria 1999	Shanghai	Community-based	70.2 ± 10.5	24.7 ± 3.3	40.5	NA	134 ± 12	80.8 ± 6.9	516	21
Xu (2016) [30]	2014-2015	CTM criteria 2010	WHO criteria 2004	Linyi, Shandong	Hospital-based	56.9 ± 9.9	NA	NA	NA	NA	NA	500	19
Zhang (2016) [31]	2011	ADA criteria 2007	WHO criteria 1999	Dalian, Liaoning	Hospital-based	61.5	25.7	NA	NA	152.9	83.23	2345	20
Li (2014)-1 [32]	2009	Clinical diagnosis	Clinical diagnosis	Tongxiang, Zhejiang	Hospital-based	>60	NA	57.7	NA	NA	NA	302	20
Li (2014)-2 [32]	2012	Clinical diagnosis	Clinical diagnosis	Tongxiang, Zhejiang	Hospital-based	>60	NA	57.7	NA	NA	NA	494	20
Zeng (2014) [33]	2010-2013	Clinical diagnosis	ADA criteria 2009	Guangzhou, Guangdong	Hospital-based	53.3 ± 13.1	NA	56.7	1~24	NA	NA	842	21
Hu (2016) [34]	2011-2012	Clinical diagnosis	WHO criteria 1999	Guangdong	Hospital-based	59 ± 12.9	NA	48.8	NA	NA	NA	4101	23
Wang (2017) [35]	2014-2015	KDOQI 2014	History of DM	Lanzhou, Gansu	Hospital-based	67.4 ± 16.9	NA	58.6	10.6 ± 7.9	NA	NA	558	21
Guo (2016) [36]	2005-2012	KDOQI 2012	WHO criteria 1999	Shanghai	Hospital-based	59.3 ± 12.3	25 ± 3.5	55.1	8.48	132 ± 17.0	79.9 ± 9.6	3301	22
Zhuo (2013) [37]	2003-2011	Renal biopsy	ADA criteria 2007	Beijing	Hospital-based	28-64	NA	61.9	2-20	NA	NA	244	22
Yang (2018) [38]	2014-2017	KDIGO guidelines 2012	History of DM	Hong Kong	Hospital-based	63.0 ± 10.8	NA	50.4	7.4 ± 6.4	131.7 ± 16.2	74.8 ± 10.2	31574	26
Duan (2019) [39]	2015-2017	Clinical diagnosis	The American Diabetes Association (ADA) 2009	Henan	Community-based	56.4 ± 13.1	24.4 ± 3.5	40.2	NA	NA	NA	2710	26
Liu (2010) [40]	2007	Clinical diagnosis	Clinical diagnosis	Multicenter (Shanghai, Chengdu, Beijing and Guangzhou)	Hospital-based	63.3 ± 10.2	NA	41.8	8.7	NA	NA	1524	26

Abbreviations: NA: not available; KDIGO: Kidney Disease Improving Global Outcomes; ADA: American Dental Association; KDOQI: Kidney Disease Outcomes Quality Initiative; CTM: Chinese Traditional Chinese Medicine Association. ^∗^Quality score of STROBE checklist.

**Table 2:** Prevalence of DN by study and design characteristics.

Subgroups	No. of studies	Prevalence estimate (%) and 95% CI	Heterogeneity *I*^2^ (%)	*P* value
Time				0.73
≤2000	3	18.7 (9.6-33.3)	98.6	
2001~2010	9	24.2 (19.0-30.4)	96.5	
>2010	11	24.0 (19.4-29.3)	98.7	
Diagnostic criteria for DN				<0.01
ADA criteria 1997	1	23.5 (19.7-27.9)	—	
ADA criteria 2007	1	15.4 (14.0-17.0)	—	
Clinical diagnosis	18	21.8 (17.2-27.2)	99.0	
CTM criteria 2010	1	18.0 (14.9-21.6)	—	
KDIGO 2012	1	29.7 (29.2-30.2)	—	
KDOQI 2007	1	18.5 (16.6-20.6)	—	
KDOQI 2012	1	27.1 (25.6-28.7)	—	
KDOQI 2014	1	39.4 (35.5-43.6)	—	
Mogensen criteria	2	9.5 (8.4-10.7)	3.4	
Renal biopsy	3	29.6 (7.9-67.3)	96.7	
Diagnostic criteria for DM				<0.01
1985 WHO/1999 China diagnostic standards	1	9.0 (7.7-10.5)	—	
ADA criteria 2005	1	18.5 (16.6-20.6)	—	
ADA criteria 2007	1	8.2 (5.4-12.4)	—	
ADA criteria 2009	2	29.3 (19.1-42.2)	97.5	
Clinical diagnosis	8	24.7 (15.5-36.9)	99.0	
History of DM	6	35.3 (30.7-40.2)	92.5	
WHO criteria 1985	2	13.9 (6.9-26.1)	97.5	
WHO criteria 1999	8	16.9 (13.4-21.2)	97.8	
WHO criteria 2004	1	18.0 (14.9-21.6)	—	
Region				<0.01
Central region	3	15.6 (4.9-39.8)	99.6	
East region	19	22.3 (18.6-26.5)	97.4	
Northeast region	3	20.7 (15.2-27.6)	96.5	
West region	2	41.3 (37.1-45.6)	39.0	
Population source				0.52
Community-based	5	18.5 (10.0-31.5)	99.0	
Hospital-based	25	22.4 (18.8-26.5)	99.1	
Age				0.15
<60	12	24.8 (20.2-30.1)	97.9	
≥60	9	19.5 (14.9-25.1)	98.8	
BMI				0.20
<25	4	14.4 (7.3-26.4)	97.7	
≥25	5	23.8 (15.4-34.8)	99.5	
Sex				<0.01
Male-dominated	16	27.7 (24.1-31.7)	97.3	
Female-dominated	10	17.6 (12.6-24.0)	99.2	
Urban and rural				0.12
Rural	2	26.2 (13.0-45.7)	99.2	
Urban	26	20.5 (17.1-24.3)	99.1	
Urban and rural	2	37.0 (21.2-56.2)	96.2	
DM duration				0.27
<8	7	26.0 (17.7-36.4)	99.6	
8~9	5	17.4 (11.2-26.1)	98.9	
10~	2	29.0 (14.1-50.4)	98.8	
Sample size				0.25
<1000	17	24.5 (18.8-31.3)	97.6	
1000~3000	9	17.3 (12.4-23.6)	98.9	
3000~	4	21.8 (13.7-33.0)	99.8	
Quality				0.47
20	5	26.3 (14.0-43.8)	98.9	
≥20	25	20.9 (17.5-24.7)	99.1	
Systolic blood pressure				0.71
≥140	2	28.7 (7.6-66.2)	97.6	
<140	4	22.5 (13.6-35.1)	99.8	
Diastolic blood pressure				0.61
≥80	3	17.0 (5.9-40.0)	97.6	
<80	4	22.5 (13.6-35.1)	99.8	

## Additional Points

*What's New?.* This is the first meta-analysis to investigate the pooled prevalence of DN among Chinese patients with type 2 diabetes mellitus (T2DM). There is geographic and gender variation of DN prevalence among T2DM patients in China. Early screening and more effective interventions targeting DN should be implemented for people with T2DM.

## Supplementary Materials

Supplementary Materials Supplement Table S1: methodological quality assessment results for the included studies. Supplement Figure S1: funnel plot of estimation of DN prevalence.
