# Supplementary material for: Prevalence of Diabetic Nephropathy among Patients with Type 2 Diabetes Mellitus in China: A Meta-Analysis of Observational Studies
Source: J Diabetes Res. 2020 Feb 3;2020:2315607. doi: 10.1155/2020/2315607 (PMC7023800; doi:10.1155/2020/2315607)
Supplement: Supplementary Materials — Supplement Table S1: methodological quality assessment results for the included studies. Supplement Figure S1: funnel plot of estimation of DN prevalence. [file 2315607.f1.docx]

**Supplement Table S1. Methodological quality assessment results for included studies**

**
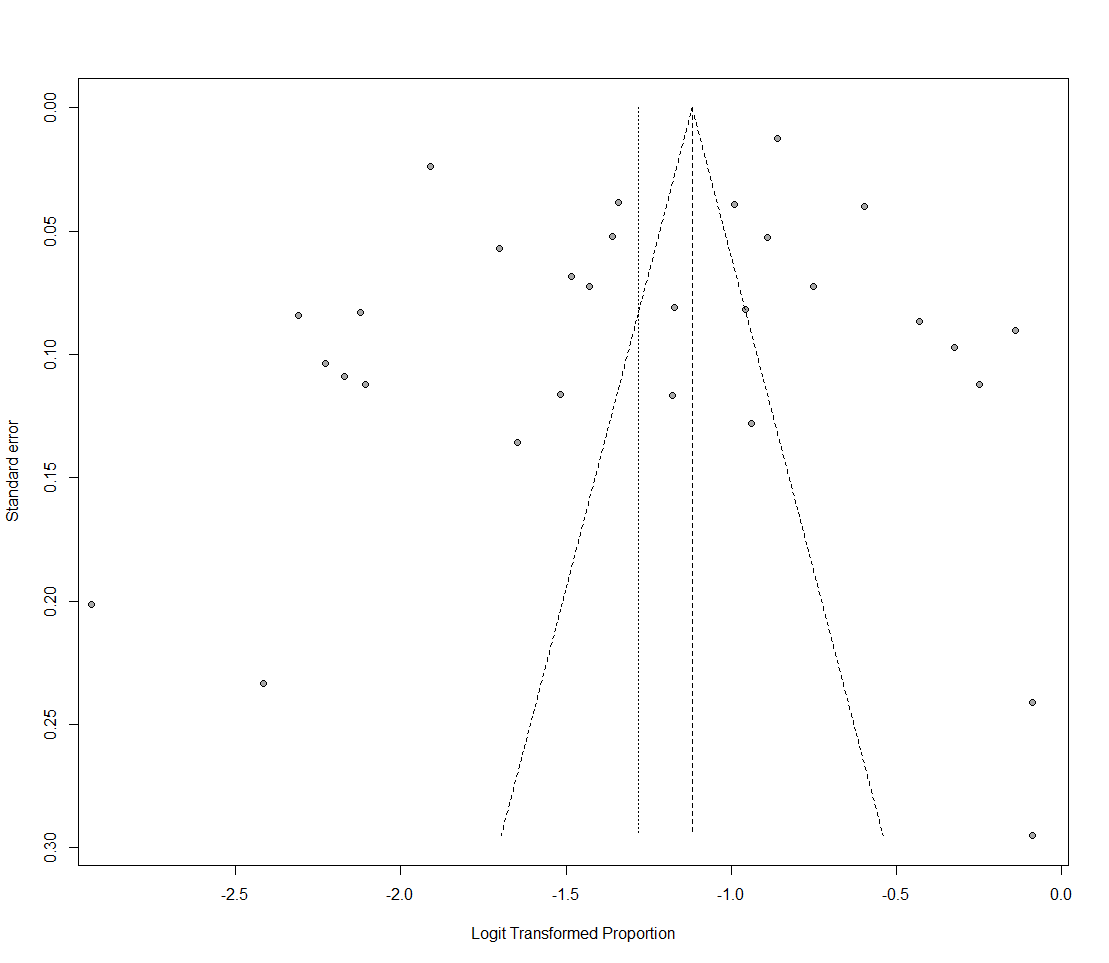
**

**Supplement Figure S1. Funnel plot of estimation of DN prevalence**
